# Weight-Loss Magnitude, Fat Mass, and Device-Estimated Muscle Mass Loss During Obesity Pharmacotherapy

**DOI:** 10.3390/jcm15208008

**Published:** 2026-10-15

**Authors:** Cristina Tomescu, Alin Albai, Vlad Florian Avram, Sandra Timar, Anamaria Cipu, Bogdan Timar

**Affiliations:** 1 Doctoral School of Medicine, “Victor Babes” University of Medicine and Pharmacy, 300041 Timisoara, Romania; cristina.tomescu@umft.ro (C.T.); anamaria.cipu@umft.ro (A.C.); 2 Centre for Molecular Research in Nephrology and Vascular Disease, “Victor Babes” University of Medicine and Pharmacy, 300041 Timisoara, Romania; avram.vlad@umft.ro (V.F.A.); bogdan.timar@umft.ro (B.T.); 3 Second Department of Internal Medicine, “Victor Babes” University of Medicine and Pharmacy, 300041 Timisoara, Romania; 4 First Department of Internal Medicine, “Victor Babes” University of Medicine and Pharmacy, 300041 Timisoara, Romania; sandra.lazar@umft.ro; 5 Multidisciplinary Research Center for Malignant Hematological Diseases (CCMHM), “Victor Babes” University of Medicine and Pharmacy, 300041 Timisoara, Romania

**Keywords:** obesity pharmacotherapy, bioelectrical impedance, device-estimated muscle mass, fat mass, weight loss

## Abstract

**Background**: Body-composition changes during routine obesity pharmacotherapy require cautious interpretation. **Methods**: This retrospective observational cohort included 59 adults with paired InBody 770 assessments: 53 receiving tirzepatide and 6 receiving semaglutide. Paired changes and relative device-estimated muscle mass loss were analyzed in all 59; component-share analyses were restricted to the 51 achieving ≥5% weight loss. Analyses used rank-based methods and exploratory robust regression. **Results**: Recorded treatment duration was a median of 7.0 months, and median observed weight loss was 12.11%. Median fat mass and device-estimated muscle mass losses were 9.9 and 2.3 kg, respectively. Among the 51 participants achieving ≥5% weight loss, median fat, device-estimated muscle, and residual shares were 73.95%, 14.91%, and 10.85%. Each additional five percentage points of weight loss was associated with 2.47 percentage points greater relative device-estimated muscle mass loss (95% CI, 1.89 to 3.05). The corresponding fully adjusted association with muscle share was −0.52 percentage points (95% CI, −1.66 to 0.62); this does not establish stability or equivalence. Final drug-specific dose intensity was not statistically associated with either outcome in the adjusted models. **Conclusions**: Weight loss observed in this selected, predominantly tirzepatide-treated cohort was mainly accounted for by estimated fat mass reduction. Larger observed weight-loss responses accompanied greater device-estimated muscle mass reductions. Muscle-share estimates were uncertain and model-dependent. The study cannot establish causal medication effects or functional consequences.

## 1. Introduction

Obesity is a chronic, relapsing disease characterized by excess or dysfunctional adiposity, and its prevalence has increased across age groups and world regions [1,2]. Because effective care must be sustained over time, treatment goals extend beyond short-term weight change to improvement in health and maintenance of the achieved response [1,2]. Incretin-based anti-obesity medications have expanded the nonsurgical weight loss attainable in randomized trials: semaglutide 2.4 mg and tirzepatide produce large mean reductions in body weight, while semaglutide has also demonstrated cardiovascular benefit in adults with established cardiovascular disease and overweight or obesity [3,4,5]. These advances have intensified interest not only in how much weight is lost, but also in which body compartments account for that loss [3,4,5].

Trial efficacy, however, does not map uniformly onto routine practice. Comparative clinical data show substantial between-person variation in achieved weight loss with semaglutide and tirzepatide, and real-world effectiveness is influenced by treatment persistence, dose escalation, dose availability, clinical indication, and patient characteristics [6,7]. Accordingly, mean weight change alone may obscure clinically relevant heterogeneity. Describing response thresholds, follow-up duration, and drug-specific dose intensity alongside achieved weight loss can help distinguish medication exposure from the biological and behavioral response ultimately observed [6,7].

Weight loss is not synonymous with fat loss. Most interventions reduce both fat mass and fat-free mass, but the proportions vary widely across individuals and interventions [8]. Baseline adiposity influences the partitioning of weight change: people with greater initial fat stores generally lose a smaller proportion of weight as fat-free tissue, although sex, age, rate and magnitude of weight loss, energy intake, and physical activity also contribute [8,9,10]. Importantly, fat-free mass and skeletal muscle mass are not interchangeable constructs; fat-free mass includes body water, organs, connective tissue, and the nonlipid component of adipose tissue, and an apparent reduction in this compartment is not necessarily equivalent to loss of contractile muscle [11]. For that reason, pharmacotherapy studies should report both absolute and relative compartment changes and should examine the fraction of total weight loss contributed by each measured component [10,11].

Evidence specific to modern incretin-based therapy generally indicates preferential fat mass loss, but the relative contribution of lean- or muscle-related compartments varies by measurement method, population, and follow-up. In the STEP 1 dual-energy X-ray absorptiometry (DXA) substudy, semaglutide reduced total fat mass and lean body mass, while lean mass increased as a proportion of body weight [12]. In the SURMOUNT-1 substudy, approximately three-quarters of weight loss with tirzepatide was fat mass and one-quarter was lean mass [13]. Systematic reviews also document heterogeneity by agent, comparator, and the compartment measured [14,15]. Whether measured changes represent adaptation to a smaller body size or clinically adverse muscle depletion cannot be determined without considering strength, performance, and muscle quality [11,16].

In routine obesity care, multifrequency bioelectrical impedance analysis is attractive for serial assessment because it is rapid, noninvasive, and widely available. Studies of the InBody 770 show good agreement or reproducibility for group-level fat and fat-free mass estimates across a broad body mass index range, while also identifying systematic offsets relative to dual-energy X-ray absorptiometry and emphasizing the need for standardized conditions and cautious interpretation of individual values [17,18]. Moreover, a device-derived muscle-mass estimate is a body-composition outcome rather than a diagnosis of sarcopenia. Contemporary consensus definitions require assessment of muscle strength and muscle quantity or quality, with physical performance used to establish severity; sarcopenic obesity additionally requires excess adiposity together with impaired muscle function [19,20]. Real-world body-composition cohorts of semaglutide and tirzepatide are comparatively limited and have reported differing patterns of muscle-mass change across settings and follow-up intervals [21,22].

The present study examined adults receiving obesity pharmacotherapy who underwent paired InBody 770 assessments. Its objectives were to describe observed weight-loss magnitude and changes in anthropometry, fat mass, and device-estimated muscle mass; estimate component shares among participants achieving at least 5% weight loss; and explore associations with weight-loss magnitude, age, sex, baseline BMI, baseline device-estimated muscle mass, recorded treatment duration, and final drug-specific dose intensity. We hypothesized that fat mass would account for most observed weight loss and examined whether larger weight reductions were accompanied by a larger device-estimated muscle share. These observational analyses were not designed to establish causal treatment effects, equivalence of component shares, or muscle function.

## 2. Materials and Methods

### 2.1. Study Design, Setting, and Reporting Framework

This retrospective observational cohort evaluated adults with overweight or obesity receiving pharmacotherapy in routine care at the Clinical Obesity Centre of the “Pius Brinzeu” Emergency Hospital, Timisoara, Romania. Data were abstracted retrospectively from clinical follow-up records during May–September 2026. This was the chart-abstraction and consent/enrollment window, not a common interval during which all participants initiated and completed treatment. Clinical assessments reflected each participant’s treatment trajectory, with recorded durations of 1–16 months. Individual calendar dates of baseline, treatment initiation, and final assessment were not included in the analytical dataset; separate date ranges for these events therefore cannot be reconstructed or reported from that dataset. Objectives were to describe observed weight loss and device-estimated compartment changes and explore their associations within the treated cohort.

Treatment was not assigned for research purposes; medication choice, dose escalation, and follow-up timing reflected routine clinical decision-making. The study contained no untreated comparator for the analyses reported in this article. Accordingly, all estimates were interpreted as within-cohort observational associations and not as causal effects of treatment, dose, or follow-up duration.

### 2.2. Participants and Analytical Cohort

Patients were identified consecutively through review of clinical follow-up records during the May–September 2026 abstraction window. The source series comprised 75 consenting patients, all with paired assessments. Sixteen diet-only records were outside the scope of this pharmacotherapy analysis. All 59 patients receiving tirzepatide or semaglutide were adults aged ≥18 years with overweight or obesity and had paired body weight and InBody 770 measurements from the same device, together with complete outcomes and model covariates. No eligible pharmacotherapy patient was excluded, and none lacked paired assessments. Figure S1 shows the source series, treatment-specific selection, and analytical subgroups.

The analytical cohort comprised 59 participants: 53 receiving tirzepatide and 6 receiving semaglutide. All 59 contributed to paired analyses and Model A. The 51 participants achieving ≥5% weight loss contributed to component-share analyses and Model B; the other eight were omitted only from share analyses. No missing analysis values were imputed. For all pharmacotherapy participants, the baseline body-composition measurement was performed on the treatment-initiation date, before the first dose. The second measurement was the available follow-up assessment used in this analysis.

### 2.3. Pharmacotherapy Exposure and Clinical Management

Treatment choice and escalation followed routine clinical decisions. The source field labeled “Maximum Dose” represented the dose at the final assessment for each participant. No dose de-escalation occurred during the observed treatment period, so the maximum recorded dose and the final dose coincided. Drug-specific dose intensity was calculated as 100 × final dose/15 mg for tirzepatide and 100 × final dose/2.4 mg for semaglutide. This is a normalized final dose, not cumulative exposure or evidence of comparable pharmacological potency across drugs.

The dataset did not contain longitudinal adherence, interruptions, adverse events, time at each dose, dietary energy or protein intake, resistance training, or habitual physical activity. These variables could not be adjusted for. Drug-specific summaries were descriptive; six semaglutide-treated participants were insufficient for a reliable between-drug comparison.

### 2.4. Anthropometric and Body-Composition Assessment

Paired body-composition measurements were obtained using the same InBody 770 multifrequency bioelectrical impedance analyzer (InBody Co., Ltd., Seoul, Republic of Korea). At both assessments, all patients were measured between 07:00 and 10:00 after an eight-hour overnight fast and after emptying the bladder. The muscle variable was the device output labeled skeletal muscle mass (SMM), reported in kilograms and referred to throughout as device-estimated muscle mass. It is not a direct anatomical measurement of skeletal muscle or an assessment of strength or performance. The retrospective analytical dataset did not include the software/firmware version, calibration or quality-control logs, or specific information on hydration and recent exercise. These details could not be verified for this analysis; full adherence to all manufacturer-recommended preparation and quality-control procedures therefore cannot be asserted.

Body weight was recorded in kilograms, BMI in kg/m^2^, body fat percentage as a percentage, and device-estimated visceral fat area in cm^2^. Waist circumference and waist-to-hip ratio were estimates produced by the InBody device, not manual tape measurements; no separately measured hip circumference was analyzed. Consequently, anatomical tape-measure landmarks, repeated manual readings, and assessor blinding for such measurements do not apply. These device-derived central-adiposity indices were interpreted as estimates rather than independent anthropometric validation. The timing covariate was recorded as treatment duration, taken directly from the source-workbook “Months” field (integer months; range, 1–16). Because baseline coincided with treatment initiation, this variable describes time on treatment from baseline, but it was not calculated as an exact day-level interval between assessments. Calendar dates and the original month-conversion/rounding rule were not available in the analytical dataset, so a days/30.44 calculation was not performed.

### 2.5. Outcomes, Response Categories, and Derived Variables

Model A used device-estimated muscle mass loss as a percentage of its baseline value. The principal explanatory variable was observed weight loss, scaled per five percentage points. Exploratory Model B used the device-estimated muscle share of total weight loss among participants achieving ≥5% weight loss. Other outcomes were absolute and relative fat mass loss, absolute device-estimated muscle mass loss, BMI, body fat percentage, and device-estimated waist circumference, waist-to-hip ratio, and visceral fat area.

Achieved weight loss (kg) = W_0_ − W_1_

Achieved weight loss (%) = 100 × (W_0_ − W_1_)/W_0_

Device-estimated muscle mass loss (%) = 100 × (M_0_ − M_1_)/M_0_

Fat mass loss (%) = 100 × (F_0_ − F_1_)/F_0_

Subscripts 0 and 1 denote the first and final assessments; W denotes body weight, M device-estimated muscle mass, and F estimated fat mass. Fat mass (kg) was calculated as body weight × body fat percentage/100, which reproduces the workbook fields labeled “Adipose Tissue Mass”. We use “fat mass” for this estimate, rather than anatomical adipose tissue mass, and reserve “lean mass” or “fat-free mass” for the distinct constructs reported in cited studies. Losses are expressed as positive magnitudes; paired-table changes are follow-up minus baseline, so reductions are negative.

Cumulative thresholds were ≥5%, ≥10%, ≥15%, ≥20%, and ≥25% observed weight loss. Mutually exclusive categories were <5%, 5% to <10%, 10% to <15%, and ≥15%. As in the submitted analysis, component shares were restricted to participants achieving ≥5% weight loss to limit small-denominator instability. This restriction defines a selected subgroup and does not remove mathematical coupling or measurement error.

Fat share (%) = 100 × (F_0_ − F_1_)/(W_0_ − W_1_)

Device-estimated muscle share (%) = 100 × (M_0_ − M_1_)/(W_0_ − W_1_)

Residual share (%) = 100 − fat share − device-estimated muscle share

The residual share is an algebraic remainder, not a separately measured tissue compartment. Device-derived estimates and body weight do not constitute an independently measured multicomponent model. Shares were not truncated in the primary analyses; an extreme muscle-share value >100% and negative residual were retained, with influence and winsorization sensitivity analyses reported separately.

### 2.6. Statistical Analysis

Categorical variables were summarized as counts and percentages. Continuous variables were summarized as medians and first and third quartiles because distribution screening identified non-Gaussian distributions, asymmetry, heavy tails, or outlying observations. Individual observations, medians, and interquartile ranges were displayed in distribution plots.

Baseline-to-follow-up changes were evaluated with two-sided paired signed-rank permutation tests using 50,000 random sign permutations. Zero differences were excluded from the signed-rank statistic, absolute-difference ties received average ranks, and differences were rounded to 10 decimal places to prevent floating-point artifacts from splitting ties. Median-change 95% percentile bootstrap confidence intervals used 6000 participant-level resamples. Benjamini–Hochberg (BH) correction was applied across the eight paired outcomes and, in that case, adjusted q values only, not unadjusted *p* values are reported.

Cumulative thresholds and mutually exclusive strata were summarized separately. Component shares were compared across the three ≥5% strata using Kruskal–Wallis H statistics and 30,000 label permutations. These comparisons and continuous share associations were exploratory and were not multiplicity-adjusted.

Monotonic associations were quantified using Spearman’s rank correlation coefficient. For associations of achieved weight loss with compartment-specific and central-adiposity reductions, 95% confidence intervals used 5000 participant-level bootstrap resamples and *p* values used 20,000 permutations. For continuous trends in component shares among responders, confidence intervals used 4000 bootstrap resamples, and *p* values used 20,000 permutations. All resampling preserved the participant as the unit of analysis.

Model A included all 59 participants; Model B included the 51 participants achieving ≥5% weight loss. Each model simultaneously included observed weight loss, age, sex, baseline BMI, baseline device-estimated muscle mass, recorded treatment duration, and final drug-specific dose intensity. Scaling was: weight loss per five percentage points, age per 10 years, BMI per 5 kg/m^2^, baseline estimated muscle mass per 5 kg, recorded treatment duration per three months, and dose intensity per 25 percentage points; female sex was the reference. In particular, Model B was exploratory: 51 observations supported seven predictors plus an intercept, with 43 nominal residual degrees of freedom. Robust fitting does not remove the risks of overfitting, ratio-outcome instability, or selection bias.

Huber M-estimation used iteratively reweighted least squares (tuning constant 1.345), with scale estimated from the median absolute deviation of centered residuals. HC3-type sandwich covariance was calculated from the final robustly weighted fit, with normal-approximation 95% confidence intervals and two-sided *p* values. These intervals are approximate because final estimated weights are treated as fixed for covariance estimation. Model coefficients describe conditional associations, not causal effects; covariate *p* values were exploratory and not multiplicity-adjusted. No equivalence margin or equivalence test was specified.

The original sensitivity analyses compared the weight-loss coefficient with ordinary least squares (OLS) using HC3 covariance, Huber regression excluding the observation with the largest Cook’s distance in the corresponding OLS fit, and Huber regression restricted to tirzepatide-treated participants. Model B was also fitted after winsorizing the outcome at the 2.5th/97.5th percentiles. Additional analyses introduced during revision compared unadjusted and age-/sex-adjusted Huber models with the full model, and estimated 95% percentile intervals by resampling participants and refitting the full Huber model in 4000 bootstrap samples. These additions were exploratory, not prespecified, and are reported in Table S1. The bootstrap resamples for Model B were drawn from the 51-participant subgroup, so inference remains conditional on that selection.

Influence in the OLS reference models was assessed using Cook’s distance, with 4/*n* considered as a conventional screening guide. This diagnostic did not define automatic exclusion. Multicollinearity was assessed using variance-inflation factors derived from auxiliary ordinary least-squares regressions. No post hoc power calculation was performed. Statistical tests were two-sided; *p* < 0.05 was used for the principal model coefficient, q < 0.05 for the family of paired outcomes, and unadjusted *p* values for exploratory secondary parameters.

Revision analyses were reproduced using Python 3.12.14, pandas 2.2.3, NumPy 2.3.5, and SciPy 1.17.0. Custom scripts implemented Huber fitting and resampling; figures were regenerated using Matplotlib 3.11.2. Fixed seeds were 20260824 for paired-outcome procedures, 20261006/20261007 for model bootstraps, 20261020–20261025 for compartment correlations, 20261030–20261032 for share correlations, and 20261040–20261042 for stratum comparisons. Resampling used participants as the unit, and permutation *p* values used a plus-one correction. Small changes from submitted resampling values reflect reproducible recalculation and explicit tie handling, not changes to participant data.

### 2.7. Ethical Considerations

The study was conducted in accordance with the Declaration of Helsinki and approved by the Institutional Review Board of “Pius Brinzeu” Emergency Hospital, Timisoara, Romania (approval no. 577, 20 October 2025). The approval covered retrospective collection of clinical data from patients who provided consent. Informed consent for use of the clinical records was obtained and documented during routine visits in the May–September 2026 consent/enrollment and chart-abstraction window. The authorized retrospective review could include earlier clinical assessments; the ethics-approval date was not the start date of every patient’s treatment.

## 3. Results

### 3.1. Pharmacotherapy Cohort and Treatment Exposure

The pharmacotherapy cohort included 59 participants with complete paired InBody 770 assessments; 53 (89.8%) received tirzepatide, and 6 (10.2%) received semaglutide. Median age was 46.0 years (IQR, 35.5 to 53.0), 16 participants (27.1%) were men, and median baseline body mass index (BMI) was 40.4 kg/m^2^ (IQR, 36.5 to 45.1). The median recorded treatment duration was 7.0 months (IQR, 4.0 to 11.0), and the median final drug-specific dose intensity was 66.7% (IQR, 33.3% to 66.7%) (Table 1).

**Figure 1:**
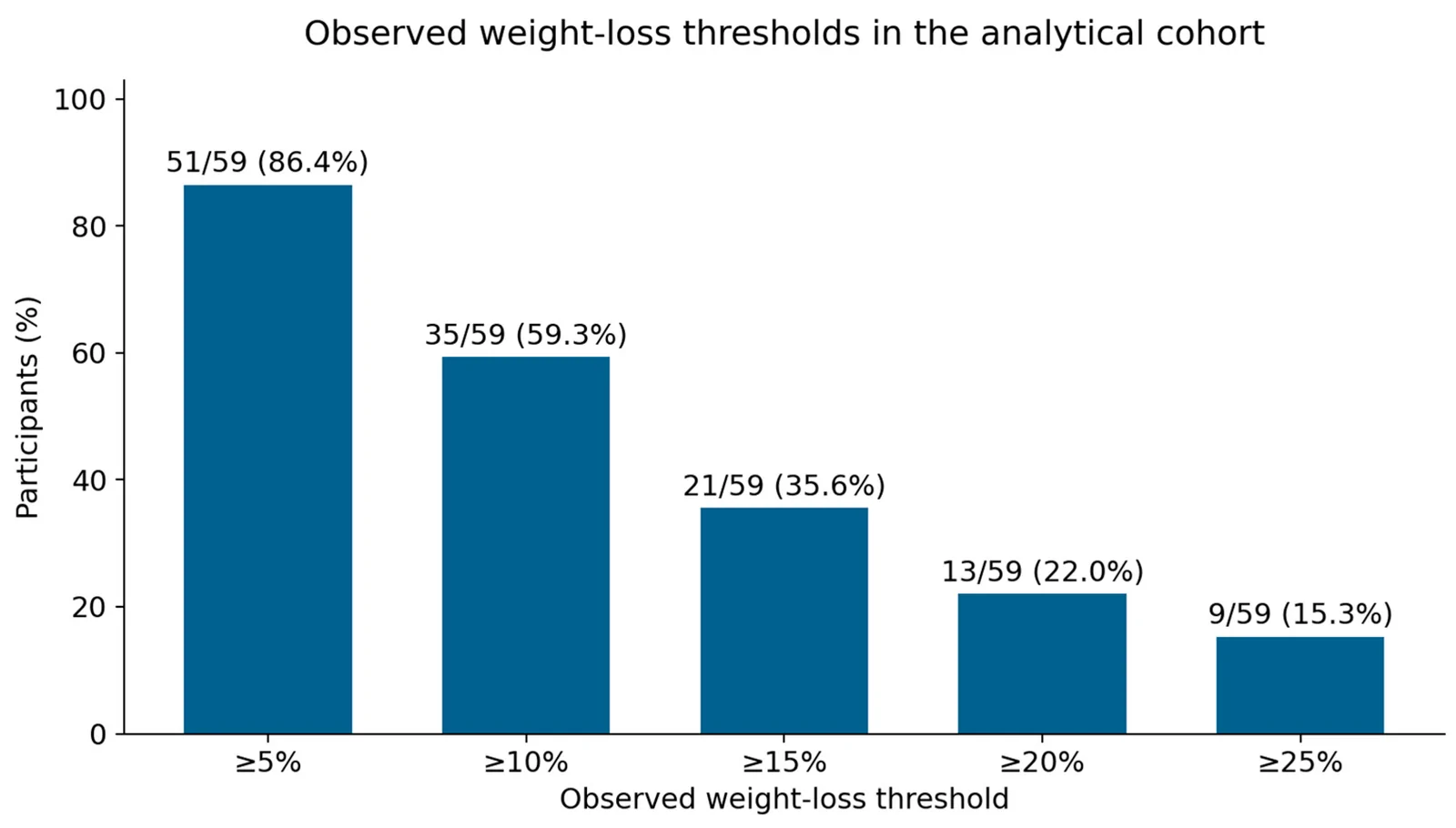
Observed cumulative weight-loss thresholds in the analytical cohort (n = 59). Each bar gives the number and percentage reaching the stated threshold.

Median achieved weight loss was 12.11% (IQR, 8.38% to 18.30%). Overall, 51 participants (86.4%) achieved at least 5% weight loss, 35 (59.3%) achieved at least 10%, 21 (35.6%) achieved at least 15%, 13 (22.0%) achieved at least 20%, and 9 (15.3%) achieved at least 25% (Figure 1).

### 3.2. Paired Anthropometric and Body-Composition Changes

**Figure 2:**
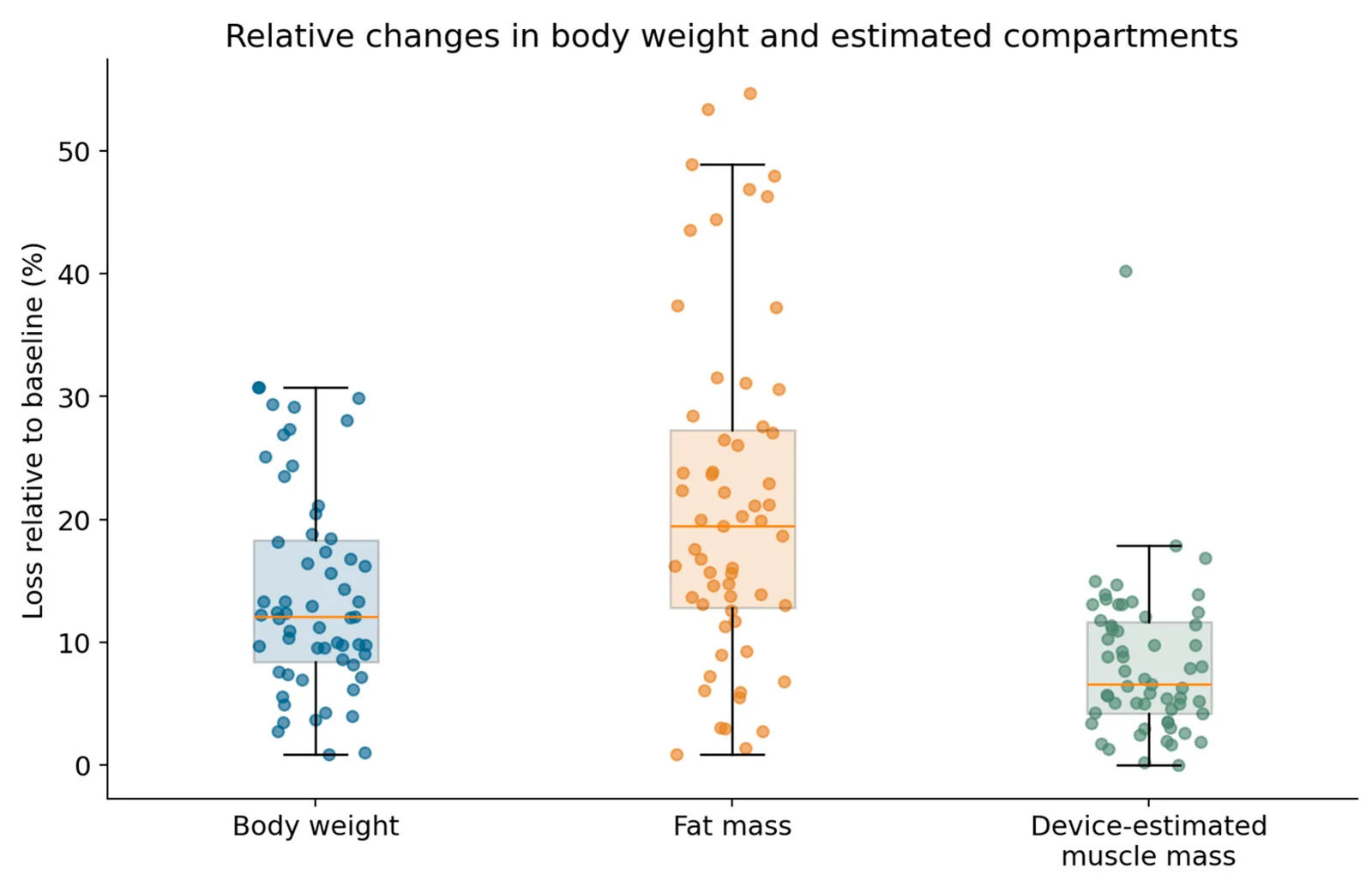
Relative reductions in body weight, fat mass, and device-estimated muscle mass (n = 59). Points denote participants, boxes show medians and interquartile ranges, and whiskers extend to the most extreme observations within 1.5 interquartile ranges.

Median paired body-weight change was −13.5 kg (IQR, −21.6 to −9.2 kg), with a median observed weight loss of 12.11%. Median fat mass loss was 9.9 kg (IQR, 6.2 to 14.9 kg), and median device-estimated muscle mass loss was 2.3 kg (IQR, 1.4 to 3.6 kg). Median relative reductions were 19.46% (IQR, 12.82% to 27.31%) and 6.62% (IQR, 4.24% to 11.63%), respectively (Figure 2).

BMI, device-estimated waist circumference, body fat percentage, and device-estimated visceral fat area decreased between assessments. All eight paired outcomes met q < 0.05 after BH correction, including device-estimated waist-to-hip ratio (median paired change, −0.02; IQR, −0.07 to 0.01; q = 0.004) (Table 2).

### 3.3. Weight-Loss Magnitude and Compartment-Specific Loss

**Figure 3:**
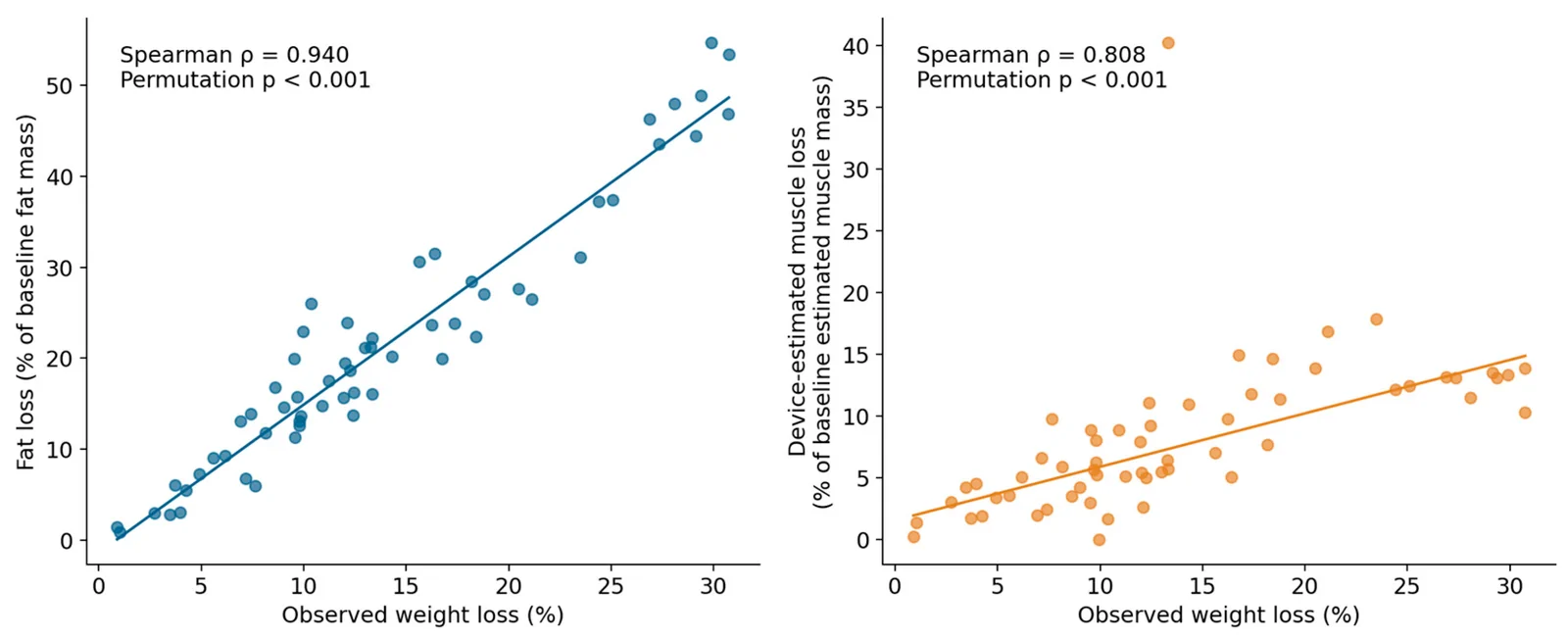
Observed weight loss and relative fat mass or device-estimated muscle mass loss. Points denote participants; lines show unadjusted Huber fits. Spearman coefficients and permutation *p* values describe monotonic associations.

Observed weight loss correlated with relative fat mass loss (Spearman ρ = 0.940; 95% bootstrap CI, 0.875 to 0.969; permutation *p* < 0.001) and relative device-estimated muscle mass loss (ρ = 0.808; 95% bootstrap CI, 0.702 to 0.869; permutation *p* < 0.001) (Figure 3, Table 3). Correlations with absolute losses were 0.940 and 0.788, respectively. Associations with device-estimated waist and visceral-fat-area reductions are shown in Table 4. These are descriptive part–whole relationships and do not identify a medication-specific mechanism.

Absolute losses of both compartments increased across achieved-weight-loss categories. Median fat mass loss rose from 1.9 kg among participants losing <5% of baseline weight to 17.1 kg among those losing ≥15%; median device-estimated muscle mass loss rose from 0.8 kg to 3.9 kg across the same categories (Table 3).

### 3.4. Component Shares Among Participants Achieving ≥5% Weight Loss

**Figure 4:**
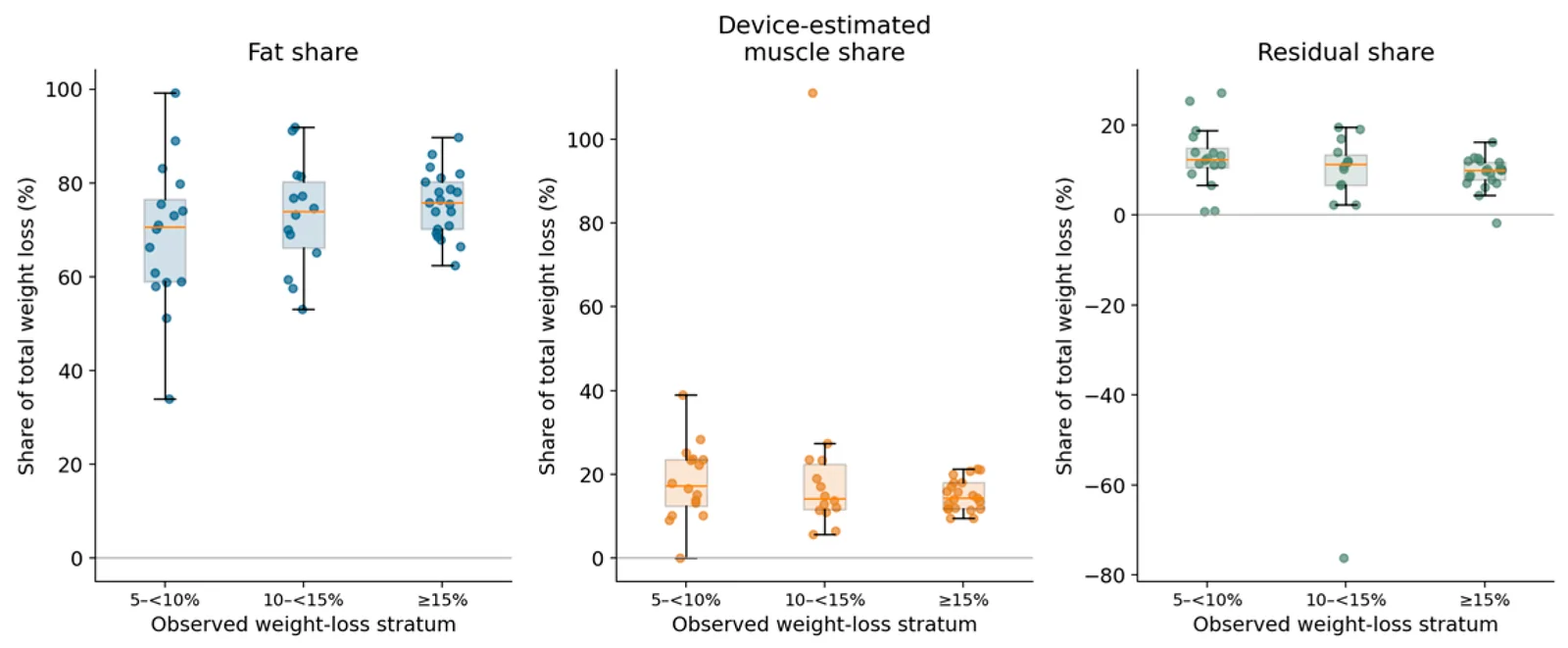
Fat, device-estimated muscle, and residual shares by observed weight-loss stratum (n = 51). Boxes show medians and interquartile ranges; points include all participants, including the extreme share and negative residual. The residual is an algebraic remainder.

Among the 51 participants achieving ≥5% weight loss, the median fat share was 73.95% (IQR, 67.21% to 80.06%), the median device-estimated muscle share 14.91% (IQR, 11.82% to 20.88%), and the median residual share 10.85% (IQR, 7.52% to 12.70%). Fat accounted for more than half of observed weight loss in 50/51 (98.0%) and at least 75% in 23/51 (45.1%); device-estimated muscle share exceeded 25% in 5/51 (9.8%). Separately calculated component medians need not sum to 100%. One retained observation had a device-estimated muscle share of 111.04% and a residual of −76.20%, illustrating the limitations of treating these device-derived changes as anatomically additive compartments (Figure 4).

Exploratory across-stratum comparisons were not statistically significant (permutation Kruskal–Wallis *p* = 0.249, 0.517, and 0.118 for fat, device-estimated muscle, and residual shares). Exploratory continuous associations were positive for fat share (ρ = 0.31; 95% bootstrap CI, 0.03 to 0.57; permutation *p* = 0.024), negative but nonsignificant for device-estimated muscle share (ρ = −0.21; 95% bootstrap CI, −0.49 to 0.09; *p* = 0.131), and negative for residual share (ρ = −0.38; 95% bootstrap CI, −0.61 to −0.10; *p* = 0.006). These unadjusted exploratory associations do not establish increasingly fat-driven responses or discrete response phenotypes (Table 5).

### 3.5. Exploratory Multivariable Associations with Device-Estimated Muscle Outcomes

**Figure 5:**
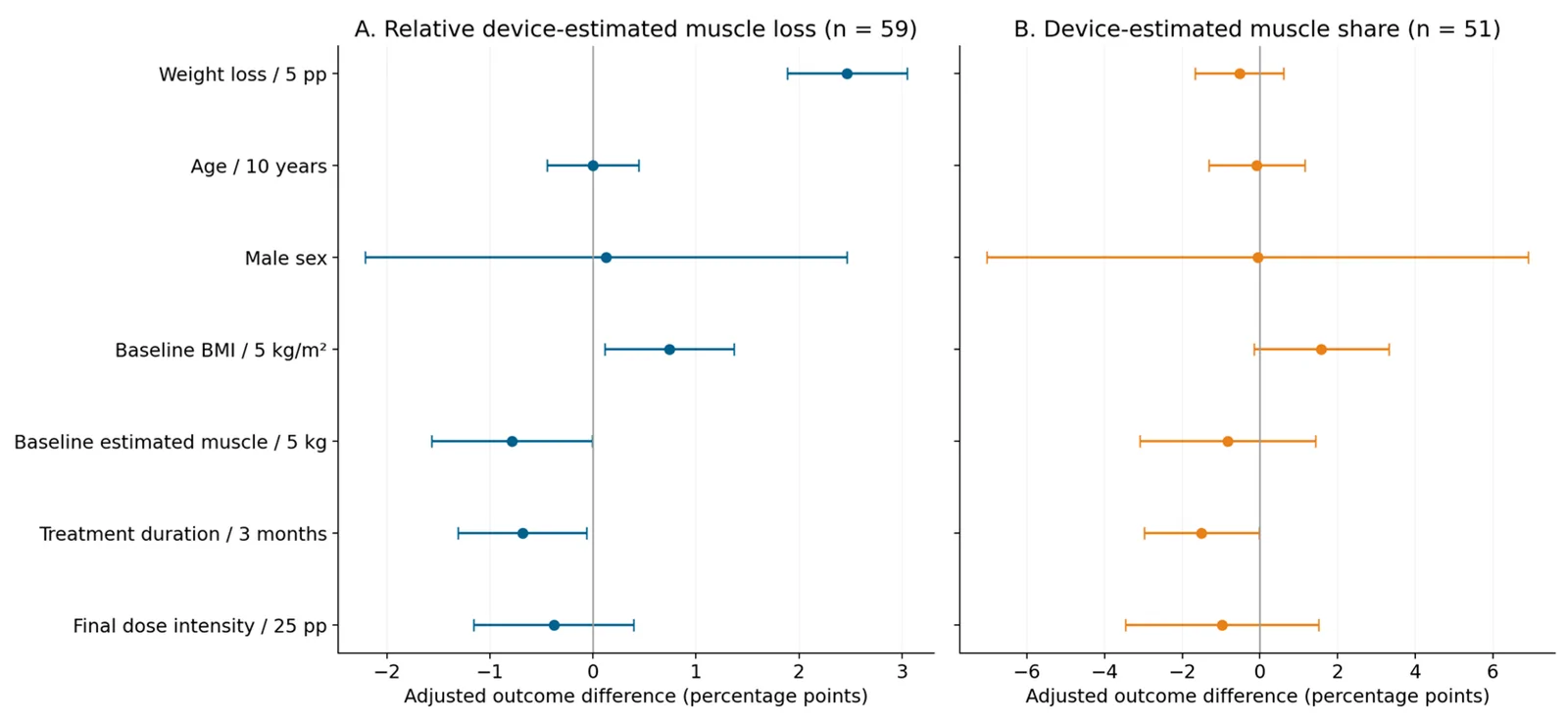
Fully adjusted Huber associations with relative device-estimated muscle mass loss and its share of total weight loss. Points are coefficients and bars are approximate HC3-type 95% confidence intervals. Model B is exploratory; its intervals do not establish equivalence.

In Model A, each additional five percentage points of observed weight loss was associated with a 2.47-percentage-point greater loss of device-estimated muscle mass relative to baseline (95% CI, 1.89 to 3.05; *p* < 0.001). In exploratory Model B, the corresponding adjusted association with the device-estimated muscle share was −0.52 percentage points (95% CI, −1.66 to 0.62; *p* = 0.368) (Figure 5). This interval is compatible with both negative and positive associations; it does not demonstrate stability, equivalence, or absence of an association.

In Model A, baseline BMI, baseline device-estimated muscle mass, and recorded treatment duration met the nominal *p* < 0.05 threshold; in Model B, only recorded treatment duration did so. Final drug-specific dose intensity was not statistically associated with either outcome in the adjusted models. This variable represents the dose recorded for the final visit rather than cumulative treatment exposure. All secondary covariate results are exploratory and uncorrected for multiplicity (Table 6).

### 3.6. Sensitivity and Influence Analyses

Across the original sensitivity analyses, the Model A weight-loss coefficient ranged from 2.44 to 2.71 percentage points per additional five percentage points of weight loss; all corresponding confidence intervals were positive. For Model B, the original sensitivity estimates ranged from −0.61 to −0.11, and all intervals crossed zero (Table 7). However, the added unadjusted and age-/sex-adjusted models gave muscle-share estimates of −1.10 (95% CI, −1.96 to −0.25) and −1.25 (95% CI, −2.20 to −0.31), respectively. Thus, inference for the share outcome depended on adjustment and should not be described as uniformly null or stable. Full-model participant-bootstrap 95% intervals were 1.87 to 3.40 for Model A and −1.92 to 1.23 for Model B (4000 valid resamples each; Table S1). Variance-inflation factors in the full models were ≤2.74; this does not establish model stability in a small sample.

## 4. Discussion

In this selected cohort with paired InBody assessments, 89.8% of participants received tirzepatide. Observed weight loss varied substantially and, among those losing ≥5%, estimated fat mass accounted for most of the reduction. Larger observed weight-loss responses accompanied larger absolute and relative device-estimated muscle mass reductions. The distinct question of whether the muscle share increased with weight-loss magnitude remained uncertain: the full-model estimate was negative with a confidence interval spanning zero, and reduced models yielded negative associations. These findings describe estimated compartment changes within treated patients; they do not identify causal medication effects, functional impairment, or preserved muscle quality.

### 4.1. Magnitude and Heterogeneity of Observed Weight Loss

Median observed weight loss was approximately 12%, with 86.4%, 59.3%, and 35.6% reaching ≥5%, ≥10%, and ≥15%, respectively. This heterogeneity is consistent with trial and routine-care evidence [3,4,6,7]. However, paired-assessment selection, variable observation intervals and doses, and unmeasured adherence and lifestyle co-interventions prevent formal effectiveness comparisons with trials or extrapolation to all patients starting treatment.

Device-estimated waist circumference and visceral-fat-area reductions correlated with weight loss, providing additional descriptive information about estimated central adiposity. Because both were outputs of the same device, their agreement with weight change is not independent anthropometric validation. These measures do not establish changes in clinical outcomes or prove mediation of cardiometabolic benefit.

### 4.2. Composition of Weight Loss in Relation to Previous Evidence

The median fat share of approximately 74% is directionally consistent with the fat predominance reported in STEP 1 and SURMOUNT-1 [12,13]. However, the approximately 15% device-estimated muscle share here cannot be equated with DXA lean-mass percentages. Lean mass and fat-free mass include components beyond skeletal muscle, and the present fat and muscle variables are device-derived estimates [10,11,23,24,25]. Measurement method, population, observation interval, and the denominator used materially affect comparisons [14,15,26].

STEP 1 reported overall DXA fat and lean changes, whereas SURMOUNT-1 also explored body-composition changes across weight-reduction tertiles [12,13]. Therefore, response-dependent partitioning is not an entirely new concept. Real-world cohorts likewise describe heterogeneity [21,22]; for example, Angelopoulos et al. studied 51 adults over 12 weeks and reported predominantly fat loss with a smaller soft-lean-mass reduction [22]. Our contribution is an exploratory routine-care analysis combining participant-level component shares with continuous weight-loss associations and influence/sensitivity assessment. It extends description to a predominantly tirzepatide-treated cohort with variable recorded treatment duration, without establishing a new biological law or superiority of one treatment.

Absolute/relative compartment loss and component share answer different questions. Because total weight change includes changes in its component tissues, a strong association between weight loss and device-estimated muscle loss is partly an expected scaling or part–whole relationship. Share analysis asks whether the estimated proportion varies, but shares also contain weight loss in their denominator, creating mathematical coupling and potential correlated error. The full-model share coefficient of −0.52 percentage points per additional five percentage points of weight loss (95% CI, −1.66 to 0.62) does not prove stability or equivalence. Negative associations in reduced models and the wider bootstrap interval further support an uncertain, model-dependent interpretation. The exploratory positive continuous fat-share association, alongside nonsignificant categorical comparisons, cannot establish that larger responses are increasingly fat-driven.

The residual share is only the remainder after subtracting estimated fat and muscle changes from weight change. It may reflect other tissues, fluid/glycogen changes, and correlated measurement error, but these contributions were not separately measured. The extreme muscle share > 100% and negative residual underline that the shares are descriptive indices, not a validated anatomical partition.

### 4.3. Clinical Context and Lifestyle Co-Therapy

Measured mass loss alone cannot distinguish physiological adaptation from impaired muscle function. The cohort lacked grip strength, chair-rise or gait testing, muscle-quality assessment, and patient-reported functional outcomes. It therefore cannot diagnose sarcopenia or sarcopenic obesity, or show that muscle loss was clinically harmless [19,20,27,28]. Body-size adaptation and potentially adverse depletion remain alternative explanations rather than conclusions [8,9,10,16,24,25,29].

As external clinical context, dietary weight-loss research supports resistance exercise and, in selected populations, adequate or supplemented protein intake for preserving lean tissue and/or strength [30,31,32,33,34,35,36,37]. Mallardo et al. discuss physical activity, nutrition, behavioral support, and individualized monitoring as co-therapy during incretin-based obesity treatment [38]. D’Alleva et al. reported body-composition and fitness improvements after 24 weeks of structured aerobic training in a small study of 13 men with obesity [39]. That pilot study is not direct evidence for resistance training during incretin therapy. Together, these sources support considering exercise, nutritional adequacy, and functional assessment in clinical care and future research; they do not demonstrate that these interventions explain or modify the changes in our cohort, where energy/protein intake and activity were not measured.

### 4.4. Multivariable Correlates, Treatment Duration, and Dose Intensity

The relative device-estimated muscle-loss association remained positive across the reported estimators and sensitivity analyses. This consistency supports a descriptive association but cannot overcome part–whole coupling or unmeasured confounding. Model B has seven predictors plus an intercept in only 51 participants, an influential observation, and a ratio outcome. Low variance-inflation factors and robust estimation do not compensate for limited information. The wider participant-bootstrap interval and adjustment-dependent share findings should temper any mechanistic interpretation.

Other covariate associations are hypothesis-generating. Normalizing muscle loss to its baseline value can mechanically associate lower baseline mass with greater relative loss and may amplify regression to the mean. Recorded treatment duration is not weight-loss velocity; its inverse coefficient could reflect clinical scheduling, response trajectories, or selection. The models cannot distinguish these explanations, and secondary covariate tests were not adjusted for multiplicity.

Final drug-specific dose intensity was not statistically associated with either outcome in the adjusted models. Final dose does not capture cumulative dose, time at each level, adherence, interruptions, or tolerability, and may itself be affected by response and clinical decisions [6,7]. Adjustment for observed weight loss also conditions on a variable that may be downstream of dose; the resulting coefficient is not the total dose–outcome association [40]. These findings neither establish biological irrelevance of dose nor support equivalence between agents.

### 4.5. Strengths and Limitations

Strengths include paired measurements using the same device, simultaneous presentation of absolute/relative change and component shares, and explicit sensitivity analyses. Reanalysis reproduced the principal regression coefficients while clarifying sampling uncertainty. These features improve transparency, but do not make the design causal or validate estimated tissue changes against a reference method.

The analysis has no untreated comparator: medication effects cannot be separated from concurrent care, behavioral changes, or time. Consecutive selection and complete paired assessments within the reviewed source series reduce missing-data concerns, but do not remove selection related to attendance, consent, or the population whose records were reviewed. The cohort is small and predominantly tirzepatide-treated; six semaglutide observations do not support medication-specific inference or generalization across anti-obesity drugs. There was no preregistration or a priori sample-size calculation.

Unmeasured energy/protein intake, resistance training, habitual activity, adherence, comorbidities, and concomitant medication may confound the findings. Baseline measurements were obtained before the first dose on the initiation date, but only two assessments and recorded treatment duration in integer months were available for analysis. Exact calendar ranges and day-level elapsed intervals could not be established from the analytical dataset. Variable treatment duration prevents evaluation of early fluid shifts, stabilization, nonlinear trajectories, or subsequent regain.

BIA depends on prediction algorithms, hydration, and body geometry. Food intake, recent exercise, fluid shifts, time of day, and incomplete preparation standardization can change estimates [17,18,41,42]. Measurements were performed in the morning after an eight-hour fast and bladder emptying, but hydration, recent exercise, device software, and calibration/quality-control documentation were unavailable for this analysis. Reusing the same device does not eliminate these influences. Waist circumference and waist-to-hip ratio were also device estimates. Agreement with DXA at group level does not make individual values interchangeable; DXA itself does not directly measure contractile muscle [43]. These measurements should therefore be interpreted as estimated body-composition changes.

Restricting share analyses to ≥5% weight loss reduces small-denominator instability but changes the target population and may introduce selection effects. Correlated measurement errors and mathematical coupling remain. Neither the full-model nonsignificant association nor the sensitivity analyses establish equivalence, stability, clinical safety, or preserved muscle function. Applicability to older/frail patients, lower-BMI populations, longer observation periods, and other medications remains uncertain.

### 4.6. Directions for Future Research

Future prospective studies should record exact assessment and treatment dates, repeated dose/adherence histories, dietary intake and exercise exposure, and measurements at common time points. Larger medication-specific cohorts with a documented screening denominator should assess component changes together with strength, performance, and patient-reported function. DXA and imaging in relevant subgroups could help distinguish device-estimated change from anatomical change [19,20,43].

Trials of individualized nutrition and structured exercise during pharmacotherapy should specify both composition and functional outcomes and follow participants through active loss and maintenance [27,28,30,31,32,33,34,35,36,37,38,39]. The present data identify questions for such studies rather than demonstrate the effects of lifestyle co-therapy.

## 5. Conclusions

In this selected observational cohort of 59 adults, 53 receiving tirzepatide, weight loss observed in the treated cohort was mainly accounted for by estimated fat mass reduction. Larger observed weight-loss responses accompanied greater absolute and relative device-estimated muscle mass reductions. These findings cannot be attributed causally to medication and should not be generalized to semaglutide or other agents.

Among the 51 participants achieving ≥5% weight loss, the fully adjusted muscle-share association was uncertain (−0.52 percentage points per additional five percentage points of weight loss; 95% CI, −1.66 to 0.62) and depended on model specification. This does not demonstrate stable shares, equivalence, or preserved muscle function. Final drug-specific dose intensity was not statistically associated with either outcome in the adjusted models; final dose is not cumulative exposure. Prospective assessment of both body composition and function is needed.

## Figures and Tables

**Table 1:** Baseline characteristics and treatment exposure in the pharmacotherapy cohort.

Characteristic	Value
Age, years	46.0 (35.5 to 53.0)
Male sex, n (%)	16 (27.1)
Initial body weight, kg	115.7 (103.5 to 125.9)
Initial BMI, kg/m^2^	40.4 (36.5 to 45.1)
Initial muscle mass, kg	31.5 (29.5 to 36.9)
Initial adipose tissue mass, kg	54.3 (47.0 to 62.9)
Initial device-estimated visceral fat area, cm^2^	240.7 (223.1 to 271.4)
Tirzepatide, n (%)	53 (89.8)
Semaglutide, n (%)	6 (10.2)
Final drug-specific dose intensity, %	66.7 (33.3 to 66.7)
Recorded treatment duration, months	7.0 (4.0 to 11.0)

Continuous variables are median (Q1 to Q3); categorical variables are n (%). Drug-specific dose intensity was calculated relative to the approved maximum dose for each drug. BMI, body mass index. Treatment duration is recorded in integer months, not recalculated from day-level dates. Muscle mass, waist-related measures, and visceral fat area are device estimates.

**Table 2:** Paired anthropometric and body-composition changes during pharmacotherapy.

Outcome	Baseline Median (IQR)	Follow-Up Median (IQR)	Change Median (IQR)	Median-Change 95% CI	BH-Adjusted q Value
Body weight, kg	115.7 (103.5 to 125.9)	98.1 (84.2 to 113.5)	−13.5 (−21.6 to −9.2)	−15.4 to −11.3	<0.001
BMI, kg/m^2^	40.4 (36.5 to 45.1)	34.6 (31.4 to 39.2)	−4.8 (−7.8 to −3.0)	−5.9 to −4.2	<0.001
Device-estimated waist circumference, cm	126.3 (116.0 to 133.4)	114.6 (103.7 to 127.4)	−9.0 (−18.1 to −4.5)	−10.4 to −6.6	<0.001
Device-estimated waist-to-hip ratio	1.04 (0.99 to 1.10)	1.04 (0.97 to 1.09)	−0.02 (−0.07 to 0.01)	−0.03 to −0.01	0.004
Muscle mass, kg	31.5 (29.5 to 36.9)	29.0 (25.8 to 35.5)	−2.3 (−3.5 to −1.4)	−3.1 to −1.9	<0.001
Body fat, %	49.3 (43.7 to 52.6)	43.8 (39.1 to 50.2)	−3.4 (−6.2 to −1.6)	−4.5 to −2.3	<0.001
Adipose tissue mass, kg	54.3 (47.0 to 62.9)	42.7 (34.5 to 52.8)	−9.9 (−14.9 to −6.2)	−12.1 to −8.5	<0.001
Device-estimated visceral fat area, cm^2^	240.7 (223.1 to 271.4)	206.8 (176.9 to 238.8)	−28.5 (−58.7 to −14.9)	−37.7 to −20.8	<0.001

Change is follow-up minus baseline; negative values denote reductions. The median paired change is calculated within participants and may differ from the difference between rounded baseline and follow-up medians. Median-change intervals are 95% percentile bootstrap confidence intervals (6000 resamples). The final column contains BH-adjusted q values only, obtained from paired signed-rank permutation *p* values (50,000 permutations), corrected across all eight outcomes. Unadjusted *p* values are not displayed. BMI, body mass index; CI, confidence interval; IQR, interquartile range. Waist circumference and waist-to-hip ratio are InBody estimates, not manual measurements.

**Table 3:** Achieved-weight-loss categories and absolute compartment losses.

Weight-Loss Category	n (%)	Weight Loss, %	Fat Mass Loss, kg	Device-Estimated Muscle Mass Loss, kg
<5%	8 (13.6)	3.6 (2.3 to 4.0)	1.9 (1.5 to 3.8)	0.8 (0.6 to 1.4)
5–<10%	16 (27.1)	8.8 (7.3 to 9.7)	6.8 (5.4 to 9.0)	1.8 (1.2 to 2.2)
10–<15%	14 (23.7)	12.3 (12.0 to 13.2)	10.0 (8.7 to 11.4)	2.3 (1.5 to 3.1)
≥15%	21 (35.6)	23.5 (18.2 to 28.1)	17.1 (14.6 to 22.4)	3.9 (3.2 to 4.1)

Values are median (Q1 to Q3) unless otherwise indicated. Categories are descriptive and mutually exclusive.

**Table 4:** Rank-based associations between observed weight loss and compartment-specific or central-adiposity reductions.

Outcome	Spearman ρ	Bootstrap 95% CI	Permutation *p*
Fat loss, kg	0.940	0.872 to 0.969	<0.001
Fat loss, %	0.940	0.875 to 0.969	<0.001
Device-estimated muscle loss, kg	0.788	0.674 to 0.858	<0.001
Device-estimated muscle loss, %	0.808	0.702 to 0.869	<0.001
Device-estimated waist reduction, cm	0.762	0.595 to 0.864	<0.001
Device-estimated visceral fat area reduction, cm^2^	0.749	0.575 to 0.859	<0.001

All analyses included 59 participants. Confidence intervals used 5000 participant-level bootstrap resamples; *p* values used 20,000 permutations. Reductions are expressed as positive magnitudes. CI, confidence interval. Waist and visceral-fat-area reductions are device-derived.

**Table 5:** Weight-loss composition by responder stratum and continuous rank-based trends.

Weight-Loss Category	n (% of 51)	Fat Share, %	Device-Estimated Muscle Share, %	Residual Share, %
5–<10%	16 (31.4)	70.6 (59.0 to 76.6)	17.2 (12.4 to 23.5)	12.4 (10.6 to 14.8)
10–<15%	14 (27.5)	73.9 (66.1 to 80.3)	14.2 (11.6 to 22.3)	11.3 (6.7 to 13.4)
≥15%	21 (41.2)	75.8 (70.2 to 80.3)	14.3 (11.8 to 18.0)	9.9 (7.9 to 11.8)

Values are median (Q1 to Q3); percentages use the 51 participants achieving ≥5% weight loss as denominator. Across-stratum permutation Kruskal–Wallis tests: fat share, H = 2.83, *p* = 0.249; device-estimated muscle share, H = 1.35, *p* = 0.517; residual share, H = 4.25, *p* = 0.118. Continuous rank associations are reported in Section 3.4 (4000 bootstrap resamples; 20,000 permutations). These analyses are exploratory and not multiplicity-adjusted. The residual is algebraically defined, and separately calculated component medians need not sum to 100%.

**Table 6:** Huber robust multivariable models of device-estimated muscle mass loss and device-estimated muscle mass share.

Predictor	Adjusted Estimate	95% CI	*p*
A. Muscle mass loss, % of baseline (n = 59)			
Achieved weight loss, per 5 percentage points	2.47	1.89 to 3.05	<0.001
Age, per 10 years	0.00	−0.44 to 0.45	0.996
Male sex	0.13	−2.21 to 2.47	0.914
Baseline BMI, per 5 kg/m^2^	0.74	0.11 to 1.37	0.020
Baseline muscle mass, per 5 kg	−0.79	−1.57 to −0.01	0.047
Recorded treatment duration, per 3 months	−0.68	−1.31 to −0.06	0.032
Final dose intensity, per 25 percentage points	−0.38	−1.16 to 0.40	0.338
B. Muscle share of total loss, % (n = 51)			
Achieved weight loss, per 5 percentage points	−0.52	−1.66 to 0.62	0.368
Age, per 10 years	−0.08	−1.32 to 1.16	0.898
Male sex	−0.06	−7.03 to 6.92	0.987
Baseline BMI, per 5 kg/m^2^	1.58	−0.16 to 3.32	0.074
Baseline muscle mass, per 5 kg	−0.83	−3.09 to 1.43	0.471
Recorded treatment duration, per 3 months	−1.50	−2.98 to −0.02	0.047
Final dose intensity, per 25 percentage points	−0.97	−3.46 to 1.52	0.445

Estimates are outcome percentage-point differences. All predictors were included simultaneously. Intervals use approximate HC3-type covariance conditional on the final Huber weights. Model B (n = 51; seven predictors plus intercept) is exploratory and does not test equivalence or stability. Covariate *p* values are not multiplicity-adjusted. Participant-bootstrap intervals and reduced models are provided in Table S1. BMI, body mass index; CI, confidence interval.

**Table 7:** Sensitivity analyses for the achieved-weight-loss coefficient.

Outcome	Analysis	n	Estimate Per 5 pp	95% CI	*p*
Muscle mass loss, %	Primary Huber	59	2.47	1.89 to 3.05	<0.001
Muscle mass loss, %	OLS-HC3	59	2.71	1.76 to 3.66	<0.001
Muscle mass loss, %	Exclude most influential observation	58	2.44	1.86 to 3.03	<0.001
Muscle mass loss, %	Tirzepatide only	53	2.47	1.79 to 3.16	<0.001
Muscle share, %	Primary Huber	51	−0.52	−1.66 to 0.62	0.368
Muscle share, %	OLS-HC3	51	−0.11	−2.30 to 2.07	0.918
Muscle share, %	Exclude most influential observation	50	−0.61	−1.66 to 0.44	0.257
Muscle share, %	Tirzepatide only	47	−0.44	−1.79 to 0.91	0.524
Muscle share, %	Winsorized at 2.5th/97.5th percentiles	51	−0.53	−1.65 to 0.58	0.347

Estimates represent the association of each additional 5 percentage points of achieved weight loss with the stated outcome. The most influential observation was identified separately for each outcome using Cook’s distance from the corresponding ordinary least-squares model. CI, confidence interval; HC3, heteroskedasticity-consistent covariance estimator; OLS, ordinary least squares; pp, percentage points.

## Data Availability

The data presented in this study is available on request from the corresponding author. The data are not publicly available due to the hospital’s privacy policy.

## Supplementary Materials

The following supporting information can be downloaded at: https://www.mdpi.com/article/10.3390/jcm15208008/s1, Figure S1, selection within the available analytical database; Table S1, additional model-sensitivity and participant-bootstrap analyses.

## Abbreviations

The following abbreviations are used in this manuscript:

BMI	Body Mass Index
CI	Confidence Interval
HC3	Heteroscedasticity-consistent Covariance Estimator Type 3
IQR	Interquartile Range
OLS	Ordinary Least Squares
VFA	Visceral Fat Area
VIF	Variance-inflation Factor
SMM	Skeletal Muscle Mass (InBody output)
BIA	Bioelectrical Impedance Analysis
