# Supplementary material for: Weight-Loss Magnitude, Fat Mass, and Device-Estimated Muscle Mass Loss During Obesity Pharmacotherapy
Source: J Clin Med. 2026 Oct 15;15(20):8008. doi: 10.3390/jcm15208008 (PMC13706178; doi:10.3390/jcm15208008)
Supplement: Supplementary file 1 [file jcm-15-08008-s001.zip › jcm-4607763-supplementary.pdf]

Figure S1. Participant selection and analytical subgroups.

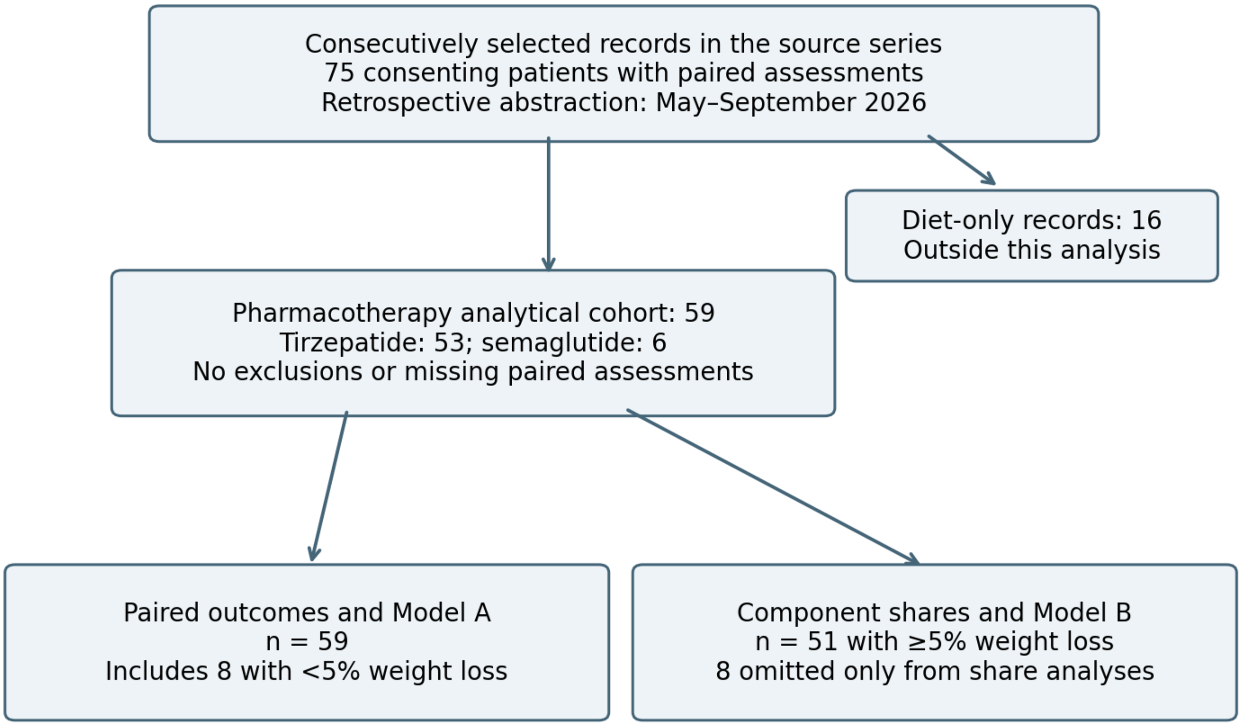

Figure S1. Selection of the pharmacotherapy cohort and analytical subgroups.
The source series refers to records selected for this retrospective study.

The consecutively selected source series comprised 75 consenting patients whose records were reviewed retrospectively during May–September 2026. All had paired assessments. Sixteen diet-only records were outside the scope of the pharmacotherapy analysis. The 59 eligible pharmacotherapy patients had complete paired measurements and analysis variables; no eligible pharmacotherapy patient in this reviewed series was excluded or lacked paired assessments. All 59 contributed to paired analyses and Model A. The 51 with ≥5% weight loss contributed to component-share analyses and Model B; the remaining eight were omitted only from share analyses.

Table S1. Additional model sensitivity and bootstrap uncertainty.

Outcome	Specification	n	Estimate	95% interval	Interval method
**Relative estimated muscle loss**	Full model	59	2.47	1.89 to 3.05	Approx. HC3-type
**Relative estimated muscle loss**	Weight loss only	59	2.17	1.88 to 2.45	Approx. HC3-type
**Relative estimated muscle loss**	Weight loss + age + sex	59	2.07	1.69 to 2.45	Approx. HC3-type
**Relative estimated muscle loss**	Full model; refit bootstrap	59	2.47	1.87 to 3.40	4000 case resamples
**Estimated muscle share**	Full model	51	-0.52	-1.66 to 0.62	Approx. HC3-type
**Estimated muscle share**	Weight loss only	51	-1.10	-1.96 to -0.25	Approx. HC3-type
**Estimated muscle share**	Weight loss + age + sex	51	-1.25	-2.20 to -0.31	Approx. HC3-type
**Estimated muscle share**	Full model; refit bootstrap	51	-0.52	-1.92 to 1.23	4000 case resamples

Each coefficient is the outcome percentage-point difference per five additional percentage points of observed weight loss. The full model includes weight loss, age, sex, baseline BMI, baseline device-estimated muscle mass, recorded treatment duration, and final drug-specific dose intensity. Reduced models intentionally estimate differently adjusted associations; they do not identify an optimal causal model. Huber fitting and predictor scaling are consistent across models. Bootstrap intervals are percentile intervals from 4000 participant-level resamples with the full model refitted in each sample (seeds 20261006 for A and 20261007 for B). All 4000 resampled design matrices had full rank for each model. Model B resamples were restricted to the 51-participant ≥5% subgroup.

The relative-loss association is positive across these specifications. The full-model share interval crosses zero, whereas reduced models yield negative intervals. Therefore, share inference is adjustment-dependent; stability, equivalence, or a uniformly absent association is not established. Bootstrap uncertainty is wider than conditional-on-final-weight covariance uncertainty. These additional analyses are exploratory and were introduced during revision.
